# Gut–Liver Axis, Microbiota, Bile Acids, and Immune Response in Pathogenesis of Primary Sclerosing Cholangitis: An Overview

**DOI:** 10.3390/jcm14217817

**Published:** 2025-11-03

**Authors:** Fotios S. Fousekis, Konstantinos Mpakogiannis, Georgios D. Lianos, Elisabetta Antonelli, Gabrio Bassotti, Konstantinos H. Katsanos

**Affiliations:** 1 Division of Gastroenterology, Department of Internal Medicine, Faculty of Medicine, School of Health Sciences, University of Ioannina, 45500 Ioannina, Greece; 2 Department of Surgery, University Hospital of Ioannina, 45500 Ioannina, Greece; georgiolianos@yahoo.gr; 3 Gastroenterology, Hepatology and Digestive Endoscopy Section, Department of Medicine and Surgery, University of Perugia Medical School, 06123 Perugia, Italy; antelibetta@yahoo.com (E.A.);

**Keywords:** primary sclerosing cholangitis, gut, microbiome, intestinal barrier, dysbiosis, inflammation, fibrogenesis, immune activation

## Abstract

Primary sclerosing cholangitis (PSC) is a chronic, immune-mediated cholestatic liver disease characterized by progressive bile duct inflammation and fibrosis. Its strong association with inflammatory bowel disease (IBD) highlights the possible role of the gut–liver axis in disease pathogenesis. Here, we review the mechanisms that may contribute to the disruption of the gut–liver axis, leading to liver injury and the development of PSC. In particular, disruption of the intestinal barrier allows microbial products to enter the portal circulation, stimulating hepatic immune cells and triggering biliary inflammation. Concurrently, gut-primed lymphocytes expressing mucosal homing receptors migrate aberrantly to the liver, where they may contribute to biliary epithelial cell injury. Dysbiosis, characterized by reduced microbial diversity and the expansion of bile-tolerant and pro-inflammatory taxa, amplifies this immune activation and disturbs gut–liver homeostasis. Moreover, bile acids act as signaling molecules, regulating metabolism and immune responses through receptors such as FXR and TGR5. Dysregulation of these pathways may promote cholestasis, inflammation, and fibrosis. By understanding these interactions, we may identify novel therapeutic targets for PSC.

## 1. Introduction

Primary sclerosing cholangitis (PSC) is a chronic, progressive cholestatic liver disease characterized by chronic inflammation and periductular fibrosis of the intrahepatic and/or extrahepatic bile ducts, resulting in biliary structures, cirrhosis, portal hypertension, and end-stage liver disease [1]. Suspicion of PSC arises from elevated cholestatic enzymes, and magnetic resonance cholangiography (MRC) is the diagnostic gold standard, revealing bile duct dilatations and strictures that form the classic beads-on-a-string pattern [2]. In addition to classic large duct PSC, there is small duct PSC, which accounts for approximately 5% of all PSC cases. Small duct PSC is a distinct clinicopathologic variant of PSC characterized by chronic cholestatic liver biochemistry and histologic features typical or compatible with PSC but with a normal cholangiogram, and its diagnosis requires a liver biopsy [3]. About 20–23% of patients with small duct PSC may progress to large duct PSC over 5–14 years [4,5]. In both large and small duct PSC, patients with may initially be asymptomatic or present fatigue, pruritus, right upper quadrant discomfort, recurrent episodes of bacterial cholangitis, or complications related to portal hypertension [6].

To date, no medical therapy has been approved. PSC is a rare disorder, with worldwide prevalence estimates in adults ranging from 0 to 16 per 100,000 and about 1.5 per 100,000 in children, and it affects all ages and races, with a predominance of males (2:1) and a peak in incidence at the age of 40 [1,7]. Despite its low prevalence in Northern Europe and the United States, it nevertheless accounts for about 10–15% of liver transplants in these regions [8]. In addition, over 30–40% of PSC patients experience recurrence after liver transplantation, sometimes requiring a second transplant [9]. Despite this burden, the underlying pathogenesis of PSC remains poorly defined, and further investigation is essential to clarify the underlying mechanisms and identify effective therapeutic targets.

An additional hallmark of PSC is its strong association with the gut, with up to 88% of PSC patients suffering from inflammatory bowel disease (IBD), particularly ulcerative colitis (UC) [10]. On the other hand, a recent meta-analysis reported a prevalence of PSC of 2.16% among patients with IBD, with rates of 2.47% in UC and 0.96% in Crohn’s disease (CD) [11]. Moreover, histological evaluation from liver biopsies has demonstrated findings compatible with PSC in up to 13% of IBD patients [12]. Patients with concomitant IBD and PSC exhibit a distinct disease phenotype, typically marked by milder intestinal inflammation but more extensive colonic involvement [13]. Additionally, patients with PSC and UC carry an increased risk of colorectal cancer compared to those with UC alone [14]. Interestingly, studies have demonstrated that a colectomy performed during or after liver transplantation can reduce the recurrence of PSC, highlighting the importance of the gut–liver axis in the progression of PSC [15,16].

**Figure 1:**
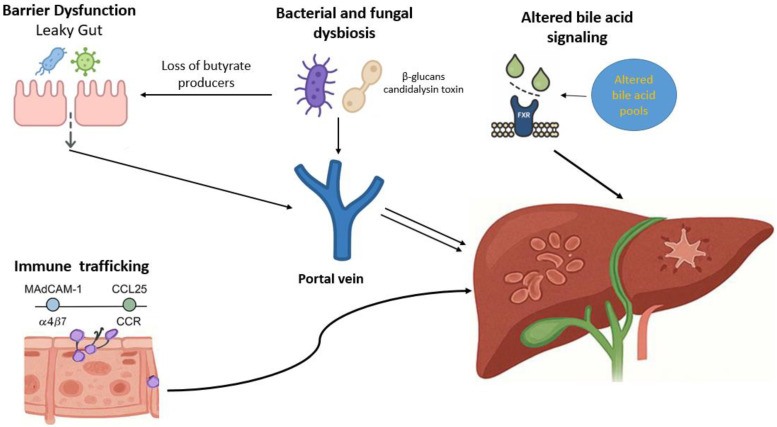
Mechanisms of gut–liver axis dysfunction in primary sclerosing cholangitis. Receptor activation disrupts bile acid homeostasis, diminishing anti-inflammatory and barrier-protective effects. Together, these mechanisms promote chronic inflammation, cholestasis, and progressive fibrosis, characteristic of PSC.

This strong association between PSC and the gut underscores the importance of the gut–liver axis in disease pathogenesis. The gut–liver axis refers to the bidirectional communication between the liver and the intestinal tract. The liver is continuously exposed to microbial products, metabolites, and immune signals derived from the intestine through the portal circulation. Homeostasis of the gut–liver axis depends on many factors, such as the integrity of the intestinal barrier, the composition and function of the gut microbiota, and the immune responses in both organs [17]. Disruption of this balanced communication may trigger pathological immune activation, bile duct injury, and fibrosis [18]. The pathophysiology of several liver diseases has been associated with dysfunction of the gut–liver axis, including non-alcoholic fatty liver disease, alcoholic liver disease, and primary biliary cholangitis [19,20]. In this comprehensive review, we aim to explore the complex interplay between the gut and the liver in PSC, focusing on key mechanisms of increased intestinal permeability, aberrant lymphocyte trafficking, altered bile acid signaling, and gut microbiota dysbiosis (Figure 1). By addressing these mechanisms, we aim to clarify how disturbances in gut–liver communication drive PSC.

## 2. Leaky Gut Hypothesis

The “leaky gut” hypothesis provides an explanation regarding how disruptions of the intestinal barrier may contribute to PSC, describing the mechanistic process of barrier breakdown and immune activation. In this model, increased intestinal permeability allows the translocation of microbial products, such as bacterial endotoxins, from the gut lumen into the portal circulation, thereby reaching the liver and triggering immune-mediated biliary injury [21]. To understand this concept, it is essential to first outline the structure and defense mechanisms of the intestinal barrier. The intestinal barrier is composed of surface mucus layers and an epithelial barrier secured by tight junctions, adherens junctions, and desmosomes, as well as underlying immune defenses. Commensal bacteria, antimicrobial peptides, and secretory IgA work together to prevent colonization by pathogens. Additionally, Paneth cells and immune cells in the lamina propria offer further protection [22,23]. Increased intestinal permeability may be caused by a combination of factors, including intestinal inflammation, such as in IBD, gut dysbiosis, genetic susceptibility, and environmental triggers [24]. Under normal conditions, the gut and liver collaborate to maintain immune system balance. Regulatory T cells (Tregs) control inflammation by moderating effector T cell activity [25]. Meanwhile, the protective barrier in the biliary system is formed by mucosal-associated invariant T cells, which express the Vα7.2-Jα33 TCR alpha chain. These cells recognize microbial vitamin B metabolites via MHC-related molecules [26,27]. They accumulate around bile ducts in the portal area, where their contact with biliary epithelial cells supports the protection of the biliary surface [28]. When exposed to bacterial products, mucosal-associated invariant T cells release interferon-γ, protecting the biliary lining. Failure of the intestinal barrier allows microbial molecules to enter the portal circulation, leading to ongoing immune stimulation in the liver. Bacterial outer membrane vesicles are able to traverse the compromised mucosal barrier and activate hepatic immune responses via Toll-like receptor 4 (TLR4) and the NLRP3 inflammasome pathway, leading to gasdermin-D-mediated pyroptosis and pro-inflammatory cytokine release [29,30]. Hepatic sinusoidal endothelial cells, Kupffer cells, and biliary epithelial cells may detect these signals, resulting in persistent activation that drives inflammation, tissue damage, and fibrosis [31]. In patients with PSC, mucosal-associated invariant T cells are reduced and functionally impaired, with diminished responses to bacterial and cytokine stimulation, contributing to altered mucosal immunity and increased susceptibility to infections [32,33].

In this context, evidence supports the hypothesis, as many studies have demonstrated, of elevated circulating markers of bacterial translocation in PSC patients (Table 1). A study assessed serum markers of bacterial translocation in 166 PSC patients compared to 100 healthy controls. Median lipopolysaccharide-binding protein and soluble CD14 were significantly elevated in PSC patients (13,662 vs. 12,339 ng/mL, *p* = 0.010; 1657 vs. 1196 ng/mL, *p* < 0.001). High soluble CD14 and lipopolysaccharide-binding protein were also associated with reduced liver transplantation-free survival (*p* < 0.001 and *p* = 0.005, respectively) [34]. Furthermore, a recent cross-sectional study investigated intestinal barrier dysfunction in patients with PSC after liver transplantation by measuring the levels of Reg3a, iFABP, zonulin, and calprotectin. The study involved a cohort of 26 patients with recurrent PSC, 87 patients with non-recurrent PSC, and 113 control subjects who underwent liver transplantation due to alcohol-related cirrhosis. The results showed that elevated levels of Reg3a were associated with the occurrence of PSC, while lower levels were linked to non-recurrence. Additionally, the incidence of recurrent PSC was found to correlate with fecal calprotectin and serum zonulin levels [35]. Another study provided a mechanistic link between gut-derived bacteria and PSC pathogenesis by identifying Klebsiella pneumoniae in the microbiota of PSC patients. *K. pneumoniae* was shown to disrupt the epithelial barrier, facilitating bacterial translocation and triggering liver inflammation. Gnotobiotic mice colonized with PSC-derived microbiota developed TH17 immune responses and hepatobiliary injury. Cultures from mesenteric lymph nodes isolated *K. pneumoniae*, *Proteus mirabilis*, and *Enterococcus gallinarum*, while a bacteria–organoid co-culture confirmed the epithelial-damaging effects of PSC-derived K. pneumonia [36]. A recent study found that targeting Klebsiella therapeutically can have beneficial effects. The research involved using a lytic phage cocktail specifically against PSC-derived K. pneumoniae. Administering the phage either orally or intravenously led to a reduction in Klebsiella levels in colonized mice and significantly decreased hepatic inflammation and cholangitis [37]. In addition, a study evaluated serologic markers of gut barrier dysfunction in 67 PSC patients (67% with IBD). The frequencies of antibodies against F-actin (AAA) IgA, AAA IgG, and AGA IgG were significantly higher in PSC than in healthy and UC controls. AAA IgA positivity independently predicted poor outcomes (HR 5.15, *p* = 0.022) and correlated with higher antimicrobial antibody frequencies and elevated intestinal fatty acid-binding protein (365 vs. 166 pg/mL, *p* = 0.011) [38].

Furthermore, several in vivo studies provide compelling evidence that bacterial inoculation or exposure to microbial products can induce hepatobiliary inflammation and injury [39]. To investigate PSC mechanisms, rats received rectal N-formyl L-methionine L-leucine L-tyrosine, a chemotactic peptide from *E. coli*. By day four, pronounced portal triad inflammation, mild hepatocyte necrosis, and dense mononuclear infiltration around small bile ducts were observed, with lymphocytes directly in contact with bile duct epithelial cells [40]. In another rat colitis model, the rectal administration of N-formyl L-methionine L-leucine L-tyrosine induced early portal infiltration by macrophages and granulocytes together with oxidative changes in bile duct cells without necrosis. By day 4, CD4^+^ and CD8^+^ T lymphocytes predominated, whereas oxidative damage had subsided [41]. In contrast, reducing microbial exposure may be beneficial. In an study with mice and rats, depleting gut bacteria, by using either germ-free methods or antibiotics, significantly decreased damage to biliary epithelial cells [42]. Overall, experimental and clinical data support the “leaky gut” hypothesis in PSC, suggesting that increased intestinal permeability and bacterial translocation lead to biliary inflammation.

**Table 1:** Major studies on intestinal barrier dysfunction (“leaky gut”) and primary sclerosing cholangitis.

Population/Model	Study Design	Methodology	Findings	Study
166 patients with PSC and 100 healthy controls	Cross-sectional cohort study	Measurement of serum biomarkers of bacterial translocation (zonulin, intestinal fatty acid-binding protein, soluble CD14, lipopolysaccharide, and LPS-binding protein)	PSC patients showed elevated soluble CD14 and LPS-binding protein compared with controls. High levels were independently associated with reduced liver transplantation-free survival.	Dhillon et al. [34]
26 PSC patients with recurrence after liver transplantation (rPSC), 87 PSC patients without recurrence (non-rPSC), and 113 post-transplant controls with alcohol-related cirrhosis	Cross-sectional study	Measurement of serological markers of intestinal barrier function (Reg3a, iFABP, zonulin, calprotectin) and generalized linear modeling to assess associations with PSC recurrence	Elevated Reg3a associated with PSC diagnosis; lower Reg3a linked to non-recurrence. rPSC incidence correlated with higher fecal calprotectin and serum zonulin levels. Suggests that impaired intestinal permeability contributes to rPSC pathophysiology.	Hlavaty et al. [35]
PSC patients and gnotobiotic mice colonized with PSC-derived microbiota	Experimental mechanistic study (human–animal translation)	Microbiota analysis, bacterial culture from mesenteric lymph nodes, and bacterial–organoid co-culture; functional assessment of epithelial barrier integrity and TH17 immune response; antibiotic intervention	Identified *Klebsiella pneumoniae* as a key pathobiont disrupting the epithelial barrier and inducing TH17-mediated hepatobiliary injury. Antibiotic treatment reduced inflammation.	Nakamoto et al. [36]
67 PSC patients (pediatric and adult; 67% with IBD, 20% with cirrhosis), 153 healthy controls, and 172 ulcerative colitis controls	Observational cohort study	Measurement of serum antibodies (AAA IgA/IgG, AGA IgA/IgG), I-FABP, LPS-binding protein, and antimicrobial antibodies (EndoCAb, anti-OMP Plus IgA) by ELISA	AAA IgA positivity identified PSC patients with worse prognosis and higher enterocyte damage (elevated I-FABP). Strongly associated with enhanced mucosal immune response to microbial antigens, indicating gut–liver axis involvement.	Tornai et al. [38]
Rats with colitis induced by rectal administration of *N*-formyl L-methionine L-leucine L-tyrosine derived from *E. coli*	Experimental animal study	Histological and electron microscopy analysis of bile ducts and hepatocytes	Portal inflammation and small bile duct injury resembling early PSC. Lymphocytes adhered directly to biliary epithelium.	Yamada et al. [40]

AAA: antibodies against F-actin; I-FABP: intestinal fatty acid-binding protein; PSC: primary sclerosing cholangitis; rPSC: recurrence of PSC after liver transplantation.

## 3. Gut Lymphocyte Homing in Liver Immunity

Regarding gut immunity, naïve lymphocytes migrate from primary lymphoid organs to secondary lymphoid tissues, including gut-associated lymphoid tissue (GALT). Within these sites, dendritic cells and other antigen-presenting cells activate them, leading to clonal expansion and the acquisition of tissue-specific homing receptors. The recruitment of lymphocytes from the bloodstream to tissues is a regulated, multistep process [43]. First, lymphocytes bind and roll along the endothelium through selectin–ligand interactions. They then respond to chemokine signals, which activate integrins to enable firm adhesion to VCAM-1 and ICAM-1 on endothelial cells, followed by transmigration into tissue microenvironments. In the gut, dendritic cells promote the expression of integrin α4β7 and CCR9 on T cells [44]. Chemokine (C-C motif) ligand 25 (CCL25) is highly specific to the small intestine within the GALT. CCL25 is constitutively and selectively expressed by small intestinal epithelial cells, and its receptor, CCR9, is found on subsets of T cells and IgA-secreting plasma cells that home to the small intestine. CCL25 is also expressed in the thymus [45,46]. These receptors enable gut-primed T cells to enter the lamina propria by interacting with MAdCAM-1, ensuring effective immune surveillance in the gut [47].

Building on this paradigm, the gut lymphocyte homing hypothesis has been proposed as another perspective on the pathogenesis of PSC. It suggests that long-living memory T cells, initially activated in a gut with possibly impaired barrier function, may travel to the liver via the portal vein and subsequently cause damage to the bile ducts. This aligns with the current understanding that PSC is a complex immune-mediated liver disease [48]. Memory T cells are long-lived, antigen-experienced T cells that recognize and fight viruses, bacteria, and cancer cells. As a result, effective memory T cells are crucial for long-lasting protective immunity and play a central role in adaptive immunity, especially in the gut and liver [49,50]. If the lymphocyte homing hypothesis is correct, memory T cells in the liver and gut should be derived from the same cell clones and/or express the same antigen.

Molecular data provide mechanistic support for this concept. In a mouse model of ovalbumin-induced colitis, T cells primed within the gut-associated lymphoid tissue by a specific antigen migrated to the liver and caused cholangitis when they recognized the same antigen on cholangiocytes [51]. A study demonstrated that the ectopic expression of CCL25 outside the gut and thymus facilitated the recruitment of CCR9^+^ T cells to the liver. CCL25 promoted the migration and α4β7-mediated adhesion of liver-infiltrating lymphocytes to MAdCAM-1, indicating the cooperative action of CCL25 and MAdCAM-1 in recruiting mucosal lymphocytes to the liver in PSC. The subsequent activation and expansion of these memory T cells may further promote the hepatic expression of CCL25 and MAdCAM-1, thereby sustaining CCR9^+^α4β7^+^ T cell recruitment and chronic inflammation, which injures the biliary epithelium [52]. Another study by Henriksen et al. also provided evidence supporting the gut lymphocyte homing hypothesis in PSC with IBD. High-throughput sequencing analyzed TCRβ repertoires from matched colon, liver, and blood samples from 10 PSC-IBD patients and paired tumor-adjacent normal gut and liver samples from 10 colon cancer patients. An average overlap of 9.7% in memory T cell clonotypes between gut and liver samples from PSC-IBD patients, excluding blood-derived clonotypes, was demonstrated, and shared clonotypes accounted for 16% of liver memory T cells and 15% of gut memory T cells. Importantly, the presence of identical T cell clones in the gut and liver suggests antigen-driven recruitment, rather than bystander trafficking [53]. Furthermore, evidence suggests that gut lymphocyte homing to the liver increases over time in later stages of PSC, worsening ongoing inflammation. In a study, liver biopsies were obtained from 20 patients with short-term PSC and from eight patients with long-term PSC, all of them having concomitant IBD. MAdCAM-1 expression was higher in the livers of patients with long-term PSC-IBD compared to controls. The proportion of CD3+ T cells expressing integrin β7 was similar in short-term PSC-IBD patients and controls, but notably higher in patients with long-term PSC-IBD [54]. Notably, another study showed that MAdCAM-1, CCL25, and E-cadherin expression was significantly higher in chronic liver disease compared to patients with a normal liver, with no differences among disease groups. The frequencies of α4β7-, αEβ7-, CCR9-, and GPR15-expressing T cells were increased in PSC-IBD and other chronic liver disease controls compared to normal tissue. These findings challenge the “gut homing” hypothesis as the main driver of PSC, suggesting that the hepatic recruitment of gut-derived T cells occurs across chronic liver diseases [55]. The mechanisms by which liver endothelial cells express gut-specific molecules remain unclear; however, it has been proposed that the upregulation of hepatic vascular adhesion protein-1 in PSC is implicated in the aberrant expression of hepatic MAdCAM-1 [56].

Although most studies have focused on T cell trafficking, new evidence indicates that B cells may follow similar pathways across the gut–liver axis, recognizing shared antigens and potentially exacerbating inflammation in PSC. Using the high-throughput sequencing of immunoglobulin heavy-chain CDR3 regions, paired gut and liver samples from 10 PSC-IBD patients were compared with those of 10 controls. PSC-IBD samples contained significantly more B cells and unique B cell clonotypes in the gut versus the liver, while antigen-specific B cell receptor sequences in control tissues were nearly absent. Importantly, 8% of B cell clonotypes overlapped between the gut and liver after excluding circulating memory clones. These shared clonotypes showed features of antigen-driven activation, including shorter CDR3 lengths and higher somatic hypermutation. These findings indicate that gut and liver B cells share a common clonal origin, supporting the role of shared antigens across the gut–liver axis in PSC [57].

## 4. The Role of Bile Acids in Gut–Liver Crosstalk

**Figure 2:**
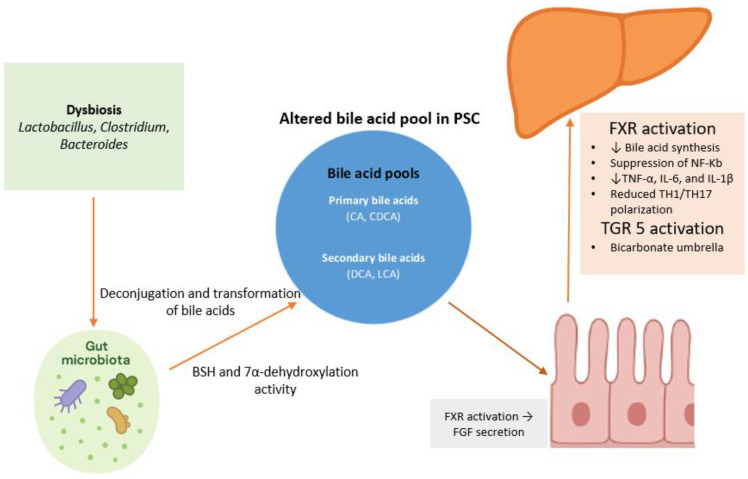
Bile acid-mediated signaling along the gut–liver axis. Bile acids from cholesterol in hepatocytes, as primary bile acids (cholic and chenodeoxycholic acid), are secreted into the intestine, where they facilitate fat absorption and undergo microbial transformation into secondary bile acids (deoxycholic and lithocholic acid). These metabolites activate nuclear and membrane receptors, primarily FXR and TGR5, expressed on enterocytes, hepatocytes, and cholangiocytes. Intestinal FXR activation stimulates FGF19 production, which suppresses hepatic bile acid synthesis through FGFR4/β-Klotho signaling, maintaining enterohepatic balance. Hepatic FXR further limits inflammation and fibrosis via NF-κB inhibition and reduced cytokine output. TGR5 activation, particularly on cholangiocytes, promotes chloride and bicarbonate secretion, forming the bicarbonate umbrella that protects the biliary epithelium. Altered microbial bile acid metabolism and receptor signaling disrupt this network, linking dysbiosis to chronic inflammation and liver injury.

Bile acids are cholesterol-derived molecules synthesized in the liver as primary bile acids, mainly cholic acid and chenodeoxycholic acid, which are conjugated and secreted into the intestine [58]. Approximately 95% of bile acids are reabsorbed in the terminal ileum via the apical sodium-dependent bile acid transporter, forming the enterohepatic circulation [59]. They appear to play a crucial role in liver metabolism, mucosal immune responses, and controlling inflammation (Figure 2). As well as their role in digesting fat, bile acids act as signaling molecules and may regulate inflammatory pathways within the gut–liver axis [60,61]. The immunomodulatory effects of bile acids are mediated through the activation of several receptors. Of these, the farnesoid X receptor (FXR) and the G-protein-coupled receptor TGR5 seem to play pivotal roles in bile acid-driven immune regulation [62].

FXR is a nuclear receptor that functions as a ligand-activated transcription factor, primarily regulating bile acid synthesis, transport, and enterohepatic circulation. FXR is highly expressed in the liver and gut and is activated by bile acids, which are its endogenous ligands [63]. Beyond bile acid regulation, FXR plays a central role in glucose, lipid, and energy metabolism, modulating genes involved in hepatic gluconeogenesis, lipogenesis, and insulin sensitivity, influencing metabolic homeostasis. Dysregulation of FXR signaling has been linked to metabolic diseases such as obesity, type 2 diabetes, non-alcoholic fatty liver disease, and non-alcoholic steatohepatitis [64,65]. Intestinal FXR activation triggers the secretion of fibroblast growth factor 19 (FGF-19), which activates hepatic FGFR4/β-Klotho receptor complexes via the portal circulation, suppressing CYP7A1 and reducing bile acid production [60,66]. This underscores the interaction between the intestine and liver. In addition, growing evidence suggests that FXR may also exert anti-inflammatory and anti-fibrotic effects in the liver, through the suppression of NF-κB activity and inhibition of cytokine production, such as TNF-α, IL-6, and IL-1β [67,68]. In this context, the pharmacological modulation of FXR, such as with the agonist obeticholic acid, is an approved therapy for primary biliary cholangitis and is under investigation for NASH [69,70]. Regarding PSC, data from animal models have demonstrated that intestinally restricted FXR agonism reduced the circulating bile acid pool, yet this alone did not improve sclerosing cholangitis. In contrast, systemic FXR activation not only suppressed bile acid synthesis but also reduced inflammatory cytokine production (IL1β, TNFα) from hepatic macrophages and limited T_H_1/T_H_17 polarization, thereby preventing the progression of hepatobiliary injury [71]. Furthermore, treatment with cilofexor—a non-steroidal FXR agonist—improved the histological features of sclerosing cholangitis, cholestasis, and hepatic fibrosis in the Mdr2^−/−^ mouse model of sclerosing cholangitis [72]. Furthermore, FXR activation has been demonstrated to be increased in the intestine and colon in PSC patients, with a two-fold rise in FXR protein levels and increased expression of target genes like OSTβ and FGF19. This may be considered as an adaptive response to chronic cholestasis and bile acid accumulation. However, despite this upregulation, PSC patients experience impaired suppression of bile acid synthesis and detoxification pathways, indicating a disconnection between FXR activation and its regulatory effects on bile acid metabolism in the liver [73].

TGR5 is expressed on biliary epithelial cells, intestinal epithelial cells, and immune cells and is activated by both primary and secondary bile acids. In the biliary tract, TGR5 activation may promote chloride and bicarbonate secretion, contributing to the so-called “bicarbonate umbrella” that shields the biliary epithelium from the detergent effects of bile acids and maintaining tight junction integrity [74,75]. Evidence from animal models suggests that the downregulation of TGR5 in biliary epithelial cells may contribute to PSC development and progression [76]. In the gut, TGR5 activation by bile acids helps to maintain epithelial barrier integrity and modulates immune responses, thereby limiting the translocation of microbial products and systemic inflammation [74]. TGR5 signaling has been found to inhibit the production of pro-inflammatory cytokines by in vitro-differentiated inflammatory and intestinal macrophages in CD [77]. Furthermore, genetic variants associated with reduced TGR5 function have been linked to an increased risk of PSC and UC, further supporting the role of TGR5 in modulating gut–liver crosstalk and immune-mediated injury [78].

In addition to FXR/TGR5 signaling, the gut microbiota plays a crucial role in forming the bile acid pool and its immunological effects. Intestinal bacteria expressing bile salt hydrolases deconjugate primary bile acids, enabling further modifications such as dehydroxylation, oxidation, and epimerization [79]. Secondary bile acids vary in hydrophobicity and receptor activity; for instance, deoxycholic acid is more pro-inflammatory, as demonstrated by its ability to induce IL-1α and IL-1β secretion and recruit neutrophils and monocytes in murine models, independently of canonical inflammasome pathways [80]. In contrast, lithocholic acid and its derivatives can activate different nuclear receptors, most notably the vitamin D receptor (VDR) and FXR [81]. This interaction between the microbiota and bile acids links dysbiosis to liver and intestinal inflammation. Altered bile acid profiles are associated with worsened non-alcoholic fatty liver disease (NAFLD), IBD, and possibly PSC by disrupting the FXR and TGR5 anti-inflammatory balance. Nevertheless, bile acids are essential in maintaining gut barrier integrity and microbial balance [60].

## 5. Microbiota Modification

The gut microbiota appears to play a crucial role in maintaining the homeostasis of the gut and liver and has also been implicated in the pathogenesis of PSC by modulating bile acid metabolism, inflammation, and the immune response [82]. Many studies have documented altered gut microbial compositions in PSC patients compared to controls, characterized by decreased alpha and beta diversity and differences in stool metabolomes [83]. Furthermore, many studies have demonstrated differences in the composition of the gut microbiota between patients with PSC and patients with PSC and IBD [84]. A cohort study analyzed the fecal microbiotas of 175 individuals using 16S rDNA sequencing. The gut microbiotas of patients with PSC were compared with those of two control groups: one comprising patients with IBD without PSC and the other comprising healthy volunteers. Patients with PSC were found to have reduced microbial diversity, with overrepresentation of the genera Enterococcus, Fusobacterium, and Lactobacillus. This dysbiosis was evident in PSC patients, with or without IBD, and was not affected by ursodeoxycholic acid treatment [85]. To overcome geographical variation, a study analyzed the gut microbiomes of individuals from two countries: Germany and Norway. They demonstrated an increase in the Proteobacteria phylum and the bile-tolerant genus Parabacteroides, which were detected independently of the geographical region. Furthermore, the associated colitis had only a minor effect on the composition of the microbiota [86]. It is worth noting that microbiota changes have been found to be present in both fecal and mucosal samples and persist after liver transplantation, suggesting a stable disease-associated microbial signature. In a cohort comprising 84 patients with PSC, 51 patients who had undergone liver transplantation, and 40 controls, 16S rRNA sequencing revealed reduced microbial diversity, with marked Proteobacteria expansion. These findings highlight the usefulness of investigating PSC and relapse PSC in parallel and suggests that the impact of the gut microbiota on post-transplant liver health should be investigated further [87]. Evidence has demonstrated that the gut microbiome in PSC may reduce the capacity for essential nutrient synthesis compared with that in healthy individuals, including vitamin B6 and branched-chain amino acids. These reductions are reflected in lower circulating levels of these metabolites in PSC patients and are associated with reduced liver transplantation-free survival [88].

Overall, PSC appears to be characterized by the increased presence of certain genera, which are often pro-inflammatory or bile-tolerant microbes, and the loss of beneficial fermenters [18]. Several studies have identified a distinct microbial profile in PSC, characterized by the increased abundance of *Veillonella* spp., *Enterococcus* spp., *Lactobacillus* spp., and *Streptococcus* spp., alongside the enrichment of members of the phylum Proteobacteria [89,90,91]. Elevated levels of the bile-tolerant genus Parabacteroides have also been reported, while sporadic increases in Fusobacteriaceae appear more prominent in PSC patients with concomitant IBD [85,86]. It is worth mentioning that an elevated Veillonella abundance is also reported in metabolic dysfunction-associated steatotic liver disease, autoimmune hepatitis, and primary biliary cholangitis [92,93,94]. This suggests that it may be a sign of advancing chronic liver disease rather than a signal specific to primary PSC.

On the other hand, a reduction in butyrate producers, namely *Prevotella Blautia* spp. and *Coprococcus* spp., has also been documented in PSC patients [89,95]. A reduction in butyrate-producing bacteria may weaken the intestinal barrier. Butyrate, a short-chain fatty acid, is a key energy source for enterocytes and plays a central role in maintaining epithelial barrier integrity. Several studies have demonstrated that butyrate may enhance tight junction protein expression, support mitochondrial function, and suppress inflammatory signaling, contributing to the integrity of the intestinal barrier [96,97].

Fungal dysbiosis is increasingly recognized as a feature of PSC, characterized by altered fungal diversity and compositions in the gut microbiotas of affected individuals. Anti-Saccharomyces cerevisiae antibodies are common in PSC patients [98]. Furthermore, bile colonization by Candida albicans is associated with a poor prognosis in these patients [99]. Lemoinne et al. demonstrated for the first time that patients with PSC exhibit fungal gut dysbiosis when compared to healthy controls. Notably, stool samples from PSC patients showed higher fungal alpha diversity, contrasting with the reduced bacterial diversity observed in these patients. Additionally, the fungal composition in PSC patients was altered, characterized by an increase in uncommon fungi, such as species from the Exophiala genus, and a decrease in Saccharomyces cerevisiae [100]. Subsequent studies have largely corroborated the presence of fungal dysbiosis in PSC. A German study found no significant increase in the alpha diversity of the mycobiota linked to PSC, but confirmed specific compositional shifts. Notably, there was overrepresentation of Sordariomycetes fungi—specifically Trichocladium griseum—and an increased abundance of Candida species in the stool of PSC patients [101]. An altered gut mycobiome, such as the overgrowth of Candida, may result in the translocation of fungal products and immune cells to the liver. Candida-reactive Th17 cells and fungal metabolites, like β-glucans and candidalysin toxin, reach the liver via the portal circulation [102,103]. In the liver, IL-17 from Th17 cells activates cholangiocytes and stellate cells, promoting inflammation and fibrosis, while β-glucans trigger IL-1β and other cytokines in Kupffer cells [104].

## 6. Conclusions

Gut–liver dysfunction appears to be a defining feature of PSC development. It is characterized by intestinal barrier failure, bacterial translocation, microbial dysbiosis, and abnormal immune activation, which lead to inflammation and fibrosis of the bile ducts (Table 2). Evidence from both experimental models and human studies supports the “leaky gut” hypothesis, demonstrating that impaired intestinal permeability facilitates the translocation of microbial products, activating hepatic Kupffer cells, the sinusoidal endothelium, and cholangiocytes. This sustained immune activation promotes cytokine release, oxidative injury, and fibrogenesis. In parallel, gut-primed lymphocytes and B cells migrate to the liver through the aberrant expression of mucosal adhesion molecules and chemokines, perpetuating antigen immune responses. Dysregulated bile acid signaling further exacerbates inflammation while compromising epithelial homeostasis. Moreover, alterations in both the bacterial and fungal composition highlight the complexity of PSC-associated dysbiosis. It should be mentioned that intestinal barrier dysfunction and microbiota alterations represent interconnected rather than independent mechanisms within the pathogenesis of PSC. Dysbiosis may weaken epithelial tight junctions and compromise mucosal integrity, facilitating translocation. All of these mechanisms support the hypothesis that PSC is a complex immune-mediated disease driven by disturbances along the gut–liver axis.

A deeper understanding of these pathways is essential in identifying possible novel therapeutic targets and developing new mechanism-based treatments for PSC. Future research in PSC should focus on integrating prospective microbiome–metabolome studies to clarify the dynamic interactions between the gut and biliary microbiota, bile acid metabolism, and host immune responses [105]. Multi-omic and longitudinal approaches using standardized sampling and analytical protocols are needed to overcome the current methodological limitations, enabling the identification of microbial and metabolomic biomarkers associated with disease activity, progression, and malignancy risk [106]. In parallel, personalized treatment approaches based on these findings should be developed, including targeted antibiotics, engineered probiotics, fecal microbiota transplantation, and bile acid receptor modulators. Ultimately, these approaches could help to classify patients by risk and guide treatments based on their microbiota, leading to the more personalized and targeted management of PSC [107].

## Figures and Tables

**Table 2:** Key gut–liver axis mechanisms implicated in primary sclerosing cholangitis.

Pathogenic Mechanism	Molecular and Cellular Components	Pathophysiological Consequences	Evidence
Intestinal barrier dysfunction	- Disruption of tight junctions (zonulin, occludin, claudins) - Immune cells, particularly Th17 lymphocytes and impaired MAIT cells - Pathobionts, such as *Klebsiella pneumonia* - Bacterial outer membrane vesicles	Increased intestinal permeability allows microbial products to reach the liver, activating Kupffer and sinusoidal cells and driving inflammation and fibrosis	[29,32,33,35,36]
Aberrant lymphocyte trafficking	- CCL25–CCR9 and MAdCAM-1–α4β7 interactions - VAP-1-dependent hepatic endothelial activation - Shared T cell and B cell clonotypes between gut and liver - Upregulation of hepatic MAdCAM-1 and CCL25	Gut-primed lymphocytes and B cells migrate to the liver via mucosal adhesion pathways, sustaining chronic biliary inflammation and immune-mediated injury	[51,52,55,56,57]
Bile acid dysregulation	- Impaired FXR and TGR5 signaling - Altered bile acid pool and secondary bile acids (DCA, LCA) - VDR activation by bile acid metabolites	Disturbed bile acid signaling and altered composition impair epithelial integrity and modulate hepatic inflammation and fibrosis	[71,76,78,81]
Bacterial dysbiosis	- Reduced microbial diversity and loss of butyrate-producing bacteria (*Prevotella*, *Blautia*, *Coprococcus*) - Enrichment of bile-tolerant and pro-inflammatory genera (*Veillonella*, *Enterococcus*, *Lactobacillus*, *Streptococcus*, *Parabacteroides*, *Proteobacteria*) - Reduced microbial capacity for vitamin B6 and branched-chain amino acid synthesis	Dysbiosis disturbs gut–liver homeostasis by weakening epithelial barrier function, altering bile acid metabolism, and enhancing mucosal and hepatic immune activation	[85,86,89,90,91]
Fungal dysbiosis	- ↑ Fungal alpha diversity - ↑ *Candida*, *Exophiala*, *Trichocladium* - β-glucans and candidalysin	Fungal components trigger Th17 and Kupffer cell activation, promoting inflammation and fibrosis	[100,101,102,103]

DCA: deoxycholic acid; LCA: lithocholic acid; FXR: farnesoid X receptor; TGR5: Takeda G-protein receptor 5; VAP-1: vascular adhesion protein-1. ↑ increased.

## Data Availability

Not applicable.
